# Diaphragmatic hernia as an infrequent complication of left pneumonectomy

**DOI:** 10.36416/1806-3756/e20240067

**Published:** 2024-08-07

**Authors:** María Emilia Cano, Fabiola Adélia Perin, Stephan Soder

**Affiliations:** 1. Departamento de Cirurgia, Pavilhão Pereira Filho, Irmandade Santa Casa de Misericórdia de Porto Alegre, Porto Alegre (RS) Brasil.

Pneumonectomy is a complex procedure, associated with significant morbidity and mortality.^1^ The most frequent complications involve the cardiovascular and respiratory systems.^2^ A 51-year-old patient underwent left pneumonectomy for lung cancer. Adhesions between the visceral and parietal pleura were found intraoperatively and dissected with monopolar electrocautery. The patient presented a successful recovery after the surgery and was discharged from hospital after nine days.

In the second month after surgery, the patient experienced a sudden onset of abdominal and thoracic pain accompanied by vomiting. The CT showed a left diaphragmatic hernia, with the presence of the stomach in the left pleural cavity. After initial measures, such as insertion of a nasogastric tube, a supra-umbilical laparotomy was performed, with satisfactory reduction of the stomach into the abdominal cavity and closure of the diaphragmatic defect with non-absorbable suture reinforced with polypropylene mesh.

Diaphragmatic hernia is a rare complication after pneumonectomy and can occur during hospital recovery or as a late postoperative complication.^3^ The iatrogenic cause is considered in this case due to the time it took for the complication to occur. The mechanism of the hernia could be a result of unintentional thinning of the diaphragm during the dissection of adhesions, which makes it more prone to rupture. The abdominal approach is recommended for the management of herniated abdominal structures, allowing correction of the diaphragmatic defect.

[Fig f1]

**Figure 1 f1:**
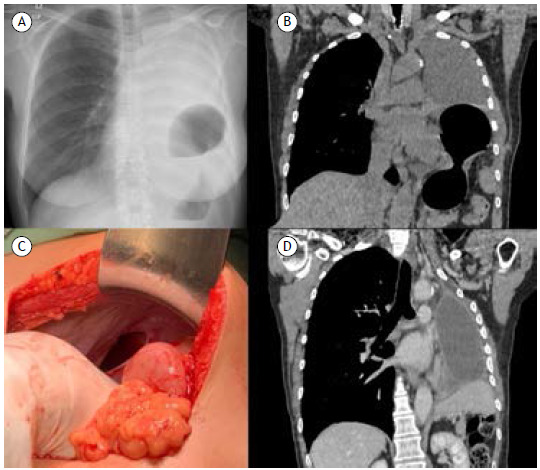
In A, a chest x-ray (frontal) performed on admission. In B., chest CT showing diaphragmatic hernia with gastric contents and pleural fluid filling the post-pneumonectomy cavity. In C, intraoperative image with identification of the diaphragmatic defect. In D, a control chest CT scan two years after diaphragmatic correction showing no herniation.
